# Pain related viral infections: a literature review

**DOI:** 10.1186/s41983-020-00238-4

**Published:** 2020-11-04

**Authors:** I. Putu Eka Widyadharma, Putri Rossyana Dewi, Ida Ayu Sri Wijayanti, Desak Ketut Indrasari Utami

**Affiliations:** Department of Neurology, Faculty of Medicine, Udayana University/Sanglah General Hospital, Bali, Indonesia

**Keywords:** Pain, Viral arthritis, Infection, Neuropathy, SARS-CoV-2

## Abstract

Pain is a common health problem all around the world. The pain symptoms are various depending on the underlying disease or the direct cause of pain itself. Viral infection could cause arthralgia or acute-onset arthritis, moreover in pandemic era of SARS-CoV-2 infection. The patients might experience arthritis, arthralgia, joint pain, or musculoskeletal pain. Viral infection including parvovirus B19, hepatitis virus, human immunodeficiency virus, arthropod-borne virus, and coronavirus could cause various types of pain. The pathogenesis of these symptoms is similar to each other despite of different causative organism. This review will discuss about pain caused by various causative organisms.

## Introduction

Pain is a common health problem in various ways and becomes a new burden of health aspects all around the world. Pain is also one of the main reasons for people to seek for doctor’s help. In recent times, minimizing the suffering and increasing quality of life are the core of medicine. Perception of pain is complex in every individual. Pain means penalty in Greek word. Plato also said that pain arises within the body and indicating that pain is more of an emotional experience. The International Association for Study of Pain (IASP) explains pain as an unpleasant sensory and emotional experience associated with actual or potential tissue damage or described in terms of such damage [1, 2]. The concept of pain affects multi-dimensional aspects of life and is related to other diseases.

The most frequently discussed nowadays is pain related to viral infection. The symptoms and signs are shown as arthritis, arthralgia, joints pain, or musculoskeletal pain. These problems arise after getting infected with some specific viruses and continue to evolve from time to time. Acute-onset arthritis is a common problem facing clinician. One percent acute-onset arthritis is caused by viral with a wide range of causal agent. The etiology of acute-onset arthritis is continuing to evolve depending on the endemic area, moreover in pandemic era of specific agent severe acute respiratory syndrome-corona virus 2 (SARS-CoV-2) [3].

The epidemiology data of incidence and prevalence of acute-onset arthritis is still lacking. Several studies showed that 1% of patients with acute-onset arthritis are caused by specific viral agent [1, 2]. Patients with viral infection sometimes show arthritis- or arthralgia-like symptoms or virally associated arthritis which is hard to distinguished. Early diagnostic and proper therapeutic should be considered as main goal of pain management in arthritis or arthralgia related to viral infections. The serological tests are believed as diagnostic tools to differentiate the causative of the acute-onset arthritis. These approaches are important for clinician in order to manage the pain caused by acute-onset arthritis and improve the quality of life for patients [2–4].

## Pathogenesis of pain related to viral infections

### Pathogenesis of pain related to the nervous systems

The pathogenesis of neuropathic pain in viral infection is still poorly understood. The processes are believed to involve the peripheral and central nervous system. Generally, a stimulus to the nociceptors will be transduced into pain impulses and transmitted by nerve fibers C and Aγ which are first-order neurons. A pain stimulus can be in the form of tissue inflammation which will cause the release of inflammatory mediators such as histamine, prostaglandin E2, and leukotriene which will stimulate nociceptors, and can also be in the form of heat, stretching, and others which also stimulate nociceptors. Pain stimuli originating from the posterior head, namely, the occipital region, ears, and neck, as well as from the dura in the posterior fossa and vertebrobasilar arteries, will be carried by the dorsal roots from the C1–C3 spinal ganglion to the dorsal horn of the spinal cord which will then sync up with neurons of the second-order and become the lateral spinothalamic tract which runs ascending and then joins with second-order neurons originating from the caudate trigeminal nucleus, then becomes the trigeminothalamic tract leading to the anteroposterior nucleus of the thalamus and synapses with third-order neurons leading to the somatosensory cortex in the post-central gyrus. Impulses coming from extracranial and intracranial pain-sensitive structures from the anterior 2/3 of the head (such as the internal and external carotid arteries and their branches such as the proximal cerebral artery, temporal artery, middle meningeal artery, and dural sinuses, trigeminal nerves V1 and V2) will be carried through the trigeminal ganglion to the trigeminus nucleus caudate/TNC or trigeminocervical complex (TCC). First-order neurons synapse with second-order neurons to form spinothalamic tracts and then become trigeminothalamic tracts to the anteroposterior nucleus of the thalamus, where they synapse with third-order neurons projecting to the primary somatosensory cortex (areas 3, 1, 2) and then to the secondary somatosensory cortex (areas 5, 7) and to the limbic system [5–7].

### Pathogenesis of pain related to other systems

The manifestation of pain in viral infection is various. It could be as acute pain or neuropathic pain based on the viral organism. Acute pain presents as arthralgia or arthritis that commonly occurs in viral infection. The pathogenesis of virally associated arthritis is still unknown. Many processes are believed to be involved in this disorder. Several studies showed that different agents could have various pathogenesis. Viral pathogen could directly invade the joints by lytic process, induce immune systems to react by immune complex formation, and also inflammatory cytokines. Otherwise, several pathogens could induce auto-immunity and inflammation by molecular mimicry, bystander activation, or epitope spreading. These complex processes may differently depend on the specific pathogen, and its way to affect the joints which will be discussed in this review [3, 4].

## Classification of viral infection differentiated by its causative organism

### Parvovirus B19

Parvovirus B19 is a member of family of single-stranded DNA viruses *Parvoviridae*. This viral infection is distributed worldwide, and nearly half of the adult population is IgG anti-B19-positive. Mostly, the cases of parvovirus B19 are asymptomatic, but on the other hand, several cases showed various clinical features including erythema infectiosum, aplastic anemia, bone-marrow suppressions in immunocompromised patients, and acute arthritis. Acute symptomatic parvovirus B19 infection is correlated with increasing numbers of pro-inflammatory cytokines. The common symptoms that occur in parvovirus B19 infection is arthralgia or acute arthritis which begins in few parts of the joint and spreads quickly after a short time. Parvovirus B19 DNA has been found in the synovial fluid of inflamed joints, suggesting immune complex involvement in arthritis related to parvovirus B19 DNA [3, 4].

The incidence of arthritis symptoms occur in various ages with 8% in children and 50–80% in adults. In children, the symptoms are distinguished as oligoarthritis affecting large joint, meanwhile in adults occur as rheumatoid arthritis (RA) which has symmetrical small joint pattern. Rheumatoid factor (RF), antinuclear antibody (ANA), and a variety of extractable nuclear antigens (ENAs) at low titers have been found in parvovirus arthritis. Mild to moderate arthritis symptoms that occur transiently or persist for several months can be treated with analgesics particularly non-steroid anti-inflammatory drugs (NSAIDs). Several severe cases of parvovirus B19 DNA infection treated with intravenous immunoglobulin (IVIG) recover successfully although further research needs to be done [3, 4, 8].

### Hepatitis B virus

Hepatitis B virus (HBV) is an enveloped double-stranded DNA virus of the *Hepadnaviridae* family which expected to affect around 350–400 million all around the world. The virus can be transmitted sexually, parenterally, vertically, or through blood-borne contact such as intravenous drug use of blood transfusion. HBV infection has a prodromal phase present 1 to 6 weeks before onset of clinical hepatitis. This symptom has been termed as arthritis-dermatitis prodrome. It could be present as arthritis or polyarthralgias with edema of joints. Arthritis related to HBV virus infection in prodrome phase varies from symmetrical and involve small joints, but also can be monoarticular and asymmetric pattern. Arthritis-dermatitis prodrome involves HBsAg-anti-HBS immune complexes. Duration of this phase around is 20 days. After the arthritis-dermatitis prodrome phase, it becomes arthritis. It is characterized by erythematous skin lesions and asymmetrical polyarthritis in chronic HBV infection.

The immune complex consists of complement components, immunoglobulin, HBsAg-anti-HBS, and inflammatory mediators, with or without RF found in synovial joint in chronic HBV infection. The definite diagnosis of HBV-associated arthritis is characterized by positive HBsAg, highly elevated aspartate aminotransferase (AST) and alanine aminotransferase (ALT), and IgM anti HBc antibodies. This symptom will relieve without any specific therapies after 2 or 3 weeks, but symptomatic treatment with analgesics can be considered if the symptom persists. The management of HBV in general is immunosuppressant in severe cases and antiviral therapy [4, 8].

### Hepatitis C virus

Hepatitis C virus (HCV) is RNA hepatotropic virus affecting around 3–4 million people annually. It can transmit like HBV infection parenterally, vertically, or sexually. Epidemiological studies showed possible role of HCV in the pathogenesis of mixed cryoglobulinemia syndrome (MCs). HCV also involves various symptoms of rheumatic and extrahepatic manifestations called HCV syndrome. Arthritis symptoms reported occur approximately in more than 70% of patients with two clinical characteristics: polyarticular small joint arthritis resembling RA and oligoarticular medium and large joint arthritis associated with MCs. The mechanism is through immune-mediated process by deposition of monoclonal RF or immune complexes in small vessel walls including synovium. Chronic HCV infection is known to stimulate the immune system and form immune complex in the vessel. The classic pathogenesis of MCs is leukocytoclastic vasculitis of capillaries, venules, and small arteries. This could be caused by deposition of immune complex in small vessel called small-vessel vasculitides, vascular deposition of HCV RNA containing cryoglobulinemia, direct viral invasion, or inflammation of perivascular. The pathologic features are degeneration of axon, vascular occlusion, and inflammation and also demyelization. It may be characterized by arthralgias, weakness, purpura, or painful peripheral neuropathy. Forty to seventy-five percent HCV patients show clinical manifestation of symptomatic peripheral neuropathy in correlation with HCV-associated MCs. According to the pathogenesis of HCV infection which can stimulate the immune system, the management of HCV infection itself is very important. The management includes eradication of the virus using interferon treatment and immunosuppressive therapy in patients with cryoglobulinemic vasculitis. Symptomatic therapies are considered as treatment of HCV-related arthritis in the absence of MCs, meanwhile MCs patients should be treated with antiviral treatment [2, 8, 9].

### Human immunodeficiency virus

Human immunodeficiency virus (HIV) infections affect the immune systems, and the number of HIV cases is increasing annually. There are approximately 700 thousand people living with HIV infection in Indonesia with 30 thousand among them are new cases of HIV infection. People with HIV infection are treated by regiment of anti-retroviral (ARV). The complications of HIV infection are various particularly 30–60% of HIV cases showed peripheral neuropathy complication. This complication related to the viral infection by two mechanisms: distal sensory polyneuropathy (DSP) and anti-nucleoside toxic neuropathy (ATN) related to their treatment [10]. The study of Parker et al. [11] showed the prevalence of pain in people living with HIV infection around 54–83% with moderate to severe intensity. Arthralgia also occur as clinical feature of pain in HIV infection. These various pain symptoms in HIV infection should be noted by the clinician in order to treat comprehensively.

### Arthropod-borne viruses

Arthropod-borne viruses (arboviruses) are group of viruses transmitted by mosquitoes. Arboviruses consist of two main viruses that related to pain or acute arthritis such as alphaviruses and flaviviruses [3, 4].

#### Alphaviruses

Alphaviruses are genus of RNA virus transmitted by mosquitoes. The common infections related to arthralgia that belong to this group are Chikungunya (CHIKV) and Ross River virus (RRV). The clinical manifestations of CHIKV basically associated with polyarthritis and rashes including prodromal symptoms like fever, headache, nausea, and myalgia [11, 12]. CHIKV infection is treated based on their symptoms. The joint symptoms in CHIKV infection occur as symmetric oligoarthralgia or polyarthralgia following fever. Arthralgia associated with CHIKV infection is often considered as a result of tenosynovitis and enthesopathy or also correlated with paresthesia symptom. These joint symptoms will resolve without any specific treatment during 1 to 4 weeks after the initial onset. Some cases lead to chronic joint pain related to CHIKV infection, but the mechanism is still unknown. NSAIDs are used to treat the joint symptoms related to CHIKV infection [11–13]. Another virus that belongs to alphaviruses is RRV. RRV is the most common mosquito-transmitted in Australia with approximately 8000 cases reported annually. RRV infection is treated regarding the symptoms. RRV also affects joint frequently, but the pathogenesis is still poorly understood. Study of Tape et al. [14] showed increased number of proinflammatory cytokine levels in RRV-related prolonged arthralgia. Analgesics or NSAID is preferable therapy for arthralgia symptom in RRV [3, 14, 15].

### Flaviviruses

Dengue virus (DENV) is a *Flavivirus* in the family *Flaviviridae* prevalent in tropical and subtropical regions in Asia, Pacific and Caribbean islands, and Central and South America. The prevalence of DENV is approximately 50–100 million cases of dengue fever (DF) annually worldwide including more than 500,000 cases of severe dengue hemorrhagic fever (DHF) and dengue shock syndrome (DSS) [16, 17]. The infection transmits by *Aedes Aegypti* mosquitoes characterized by fever, rash, myalgia, and headache. These wide clinical manifestations are similar to other Arbovirus infections such as CHIKV. In the study by Goupil et al. [15], rheumatoid arthritis-like illness was more common in DENV and CHIKV co-infection. Arthralgia is a common symptom found in DENV, meanwhile arthritis and synovitis are rare in mono DENV patients. The management of DENV infection that is symptomatic is to particularly regulate the body fluid to prevent shock at the acute phase as the pathogenesis of DENV infection is plasma leakage [16, 17].

### Herpes zoster

Herpes zoster (HZ) also known as shingles is caused by reactivation of latent varicella zoster virus (VSV). This infection often occurs in adult, and the risk is increasing by older age, approximately half of cases is older than 60 years old. The clinical manifestation of herpes zoster is painful blistering skin eruption according the dermatome of nerve. The virus remains dormant in the spinal dorsal root ganglia and ganglia of sensory cranial after the primary infection and travel along the sensory nerve as the virus reactivates caused by immunocompromised condition. As the result, the pain symptom will develop according to nerve dermatome followed by blistering skin eruption. HZ is typically unilateral according to the sensory dermatome. The challenging management of HZ is post herpetic neuralgia (PHN) complication. PHN is defined as the pain that persist at least 3 months after the rash has resolved. Damage of sensory nerves lead the neuropathic pain that occurs intermittent and presents as hyperalgesia or allodynia. Treatment of HZ is antiviral with certain dosage, corticosteroids, and pain medications. A variety of PHN therapy has been discussed, but still poorly understood. The management of PHN includes topical agents, antidepressants, specific anticonvulsants against neuropathic pain, or opioid agents [18, 19].

### Coronaviruses

Coronaviruses are the largest viruses with a positive-sense single-stranded-RNA genome, member of *Coronaviridae* family. Coronaviruses are known worldwide with a new novel coronavirus named severe acute respiratory coronavirus 2 syndrome (SARS-CoV-2) after preceded by severe acute respiratory coronavirus syndrome (SARS-CoV) and Middle East coronavirus syndrome (MERS-CoV). The phylogenetic analysis showed SARS-CoV-2 is different from SARS-CoV-1 [20, 21].

SARS-CoV-2 is a pathogen which caused coronavirus disease 2019 (COVID-19). This infection was first identified in Wuhan, China, and spread contagiously all around the world. SARS-CoV-2 is believed to be spread human to human through droplet and close contact. Several studies showed this virus has *reproductive numbers* (Ro) > 2 (2.2–2.6), means the virus could spread from 1 person to other 2.2 person. This Ro is higher than SARS-CoV-1 and MERS-CoV which is only < 1. These studies explain how contagious SARS-CoV-2 is [22, 23].

COVID-19 has various clinical manifestations including neurological manifestations. Neurological manifestations could appear as pain caused by skeletal muscle injury related to SARS-CoV-2 infection. Skeletal muscle injury leads to pain symptoms in COVID-19 patients. This clinical feature is believed to be caused by invasion of virus in the muscle and joint, inducing inflammatory response like general viral infection caused by another organism. The increasing numbers of inflammatory mediators also involve in this pathogenesis and could damage the muscle. The COVID-19 patients often experience arthralgia, muscular aches, or general weakness caused by the impact of the virus itself. The other potential mechanism of pain induced by SARS-CoV-2 in COVID-19 is SARS-CoV-2 detected in cerebrospinal fluid of infected patients. The other hypotheses are SARS-COV-2 can invade into the central nervous system. Generally, the role of angiotensin-converting enzyme 2 (ACE2) receptor in the nervous system is not fully known, although some research showed that ACE2 was detected in neuron and microglia in the spinal dorsal horn. SARS-COV-2 can affect directly or indirectly to the tissue damage including joint and muscle resulting pain in infected patients. The other mechanism SARS-COV-2 can cause is the imbalance of ACE2 and angiotensin II in the spinal cord and cause neuropathic pain. Reports of neurological sequelae after COVID-19 infection is increasing by the time associated with the involvement of ACE2 receptor in the neural tissue. Based on these reports, COVID-19 infection could manifest as painful symptoms that require pain medications [24, 25].

Creatine kinase levels might be increased above 200 U/L, increasing D-dimer, C-reactive protein (CRP), and lowered lymphocyte count if the skeletal muscle injury occurred despite of positive COVID-19 *polymerase chain reaction* test. The management of this manifestations is similar with another viral infection: symptomatic therapy depends on the symptoms occurred in patients. Further research is needed to understand how the SARS-CoV-2 could damage and impact musculoskeletal systems [24, 25]. There is no specific treatment to treat COVID-19 as the researches for this infection is still on going. Several studies showed COVID-19 infection is treated based on their symptoms. The principle to treat this infection is immunomodulator agent considered at acute or early phase, meanwhile immunosuppressant is considered at advanced phase of this infection [26, 27].

## Conclusion

Viral infection could cause various clinical manifestations including pain. There are several viral infections including parvovirus B19 virus, hepatitis B and C virus, human immunodeficiency virus, arthropod-borne virus, and coronaviruses that lead to pain symptoms. Symptomatic therapy is preferably used to treat pain-related viral infections.

## Data Availability

Not applicable.

## Abbreviations

IASP: The International Association for Study of Pain
SARS-CoV-2: Severe acute respiratory syndrome-corona virus 2
TCC: Trigeminocervical complex
RA: Rheumatoid arthritis
RF: Rheumatoid factor
ANA: Antinuclear antibody
ENAs: Extractable nuclear antigens
NSAIDs: Non-steroid anti-inflammatory drugs
IVIG: Intravenous immunoglobulin
HBV: Hepatitis B virus
AST: Aspartate aminotransferase
ALT: Alanine aminotransferase
HCV: Hepatitis C virus
MCs: Mixed cryoglobulinemia syndrome
HIV: Human immunodeficiency virus
ARV: Anti-retroviral
DSP: Distal sensory polyneuropathy
ATN: Anti-nucleoside toxic neuropathy
Arboviruses: Arthropod-borne viruses
CHIKV: Chikungunya
RRV: Ross river virus
DF: Dengue fever
DHF: Dengue hemorrhagic fever
HZ: Herpes zoster
VSV: Varicella zoster virus
PHN: Post-herpetic neuralgia
SARS-CoV: Severe acute respiratory coronavirus syndrome
MERS-CoV: Middle East coronavirus syndrome
COVID-19: Coronavirus disease 2019
ACE2: Angiotensin converting enzyme 2
CRP: C-reactive protein
